# Apoptosis Signaling Is Altered in CD4^+^CD25^+^FoxP3^+^ T Regulatory Lymphocytes in Pre-Eclampsia

**DOI:** 10.3390/ijms13066548

**Published:** 2012-05-29

**Authors:** Dorota Darmochwal-Kolarz, Shigeru Saito, Jacek Tabarkiewicz, Bogdan Kolarz, Jacek Rolinski, Bozena Leszczynska-Gorzelak, Jan Oleszczuk

**Affiliations:** 1 Department of Obstetrics and Perinatology, Medical University of Lublin, Lublin 20-950, Al. Raclawickie 1, Poland; E-Mails: b.leszczynska@umlub.pl (B.L.-G.); jan.oleszczuk@umlub.pl (J.O.); 2 Department of Obstetrics and Gynecology, University of Toyama, 2630 Sugitani, Toyama 930-0194, Japan; E-Mail: s30saito@med.u-toyama.ac.jp; 3 Department of Clinical Immunology, Medical University of Lublin, Lublin 20-950, Al. Raclawickie 1, Poland; E-Mails: kolgen@wp.pl (J.T.); bogdank@mp.pl (B.K.); jacek.rolinski@gmail.com (J.R.)

**Keywords:** apoptosis, Bax protein, Bcl-2 protein, CD95 antigen, pre-eclampsia, pregnancy, Treg cells

## Abstract

The aim of our study was to estimate the surface expressions of CD95 (APO-1/Fas) antigen and the intracellular expressions of anti-apoptotic protein Bcl-2 and pro-apoptotic protein Bax in CD4^+^CD25^+^FoxP3^+^ T regulatory lymphocytes (Tregs) as well as the percentage of CD8^+^CD28^+^ T cytotoxic cells in peripheral blood of patients with pre-eclampsia in comparison with healthy pregnant women in the third trimester of physiological pregnancy. Twenty-four women with pre-eclampsia and 20 normal third trimester pregnant women were included in the study. The lymphocytes were isolated from peripheral blood samples and labeled with monoclonal antibodies. The expressions of surface antigens and intracellular proteins were estimated using flow cytometry. The population of CD4^+^CD25^+^FoxP3^+^ Treg cells was significantly lower in peripheral blood of patients with pre-eclampsia when compared to normal third trimester pregnant women. The percentages of CD4^+^CD25^+^FoxP3^+^ Treg cells that express Bcl-2 protein were significantly lower in peripheral blood of patients with pre-eclampsia when compared to healthy pregnant women, whereas the percentages of CD4^+^CD25^+^FoxP3^+^ Treg cells with the expressions of Bax protein did not differ in both groups. Moreover, the mean fluorescence intensity (MFI) of Bcl-2 protein in CD4^+^CD25^+^FoxP3^+^ Treg cells was significantly lower and MFI of Bax protein significantly higher in pre-eclampsia when compared to the control group. The percentage of CD8^+^CD28^+^ T cells did not differ in both studied groups but MFI of CD28 antigen on T CD8^+^ cells was significantly higher in pre-eclampsia when compared to the control group. The obtained results suggest that the deficit of CD4^+^CD25^+^FoxP3^+^ Treg lymphocytes which is observed in pre-eclampsia may be associated with altered apoptosis signaling in Tregs.

## 1. Introduction

Pre-eclampsia (PE) is a common obstetric syndrome affecting about 5–10% of pregnant women [1]. The etiology and pathogenesis of this syndrome are not fully understood. There are many studies describing alterations in the innate and adaptive immune system which may have an influence on the onset of this disorder. It was suggested that the activation of cell-mediated immunity may play the key role in the etiology of pre-eclampsia. It was proposed that inappropriate activation of the immune system can lead to pre-eclampsia [1–4].

Regulatory T lymphocytes CD4^+^CD25^bright^ (Tregs) are known to play an important role in the development and maintenance of tolerance in peripheral tissues [5,6]. It has been demonstrated that Treg cells have a role in induction of transplantation tolerance. They express high level of CD25 (IL-2Rα) as well as cytotoxic T lymphocyte antigen 4 (CTLA-4) and the transcription factor Foxp3 [5–10].

Maternal immune system responses to fetal antigens play a critical role [11]. There is some evidence that T regulatory lymphocytes (Treg cells) play an essential role in controlling and preventing fetal rejection. It was suggested that high levels of Treg cells are extremely important for successful pregnancy [12–15]. Furthermore, was observed that the populations of peripheral blood and decidual Treg cells are reduced in pre-eclampsia [16–19]. On the other hand, there were reports that the numbers of peripheral blood Tregs were similar in pre-eclampsia and normal pregnancy [20,21]. However, it should be taken into account that in those studies the sample numbers were small or the authors estimated CD4^+^CD25^+^ T cells, and they did not evaluate CD4^+^CD25^high^ Treg cells [20,21].

Treg cells cooperate with CD8^+^CD28^−^ T lymphocytes which have also a regulatory capacity [22]. On the other hand, the expressions of CD28 antigens on CD8^+^ T cells enhance a T cell activation which may lead to immune responses at the fetal-maternal interface [23].

Apoptosis (programmed cell death) is an important immunoregulatory mechanism for maintaining homeostasis in the immune system. There are a few pathways controlling programmed cell death. The protein Bcl-2 is one of the factors which regulates apoptosis [24,25]. In humans the family of apoptotic proteins consists of more than 15 members divided into two groups: anti-apoptotic and pro-apoptotic proteins. The proteins: Bcl-2 and Bcl-xLong are characterized as anti-apoptotic proteins thus preventing cell death. The proteins: Bax, Bak, Bad, Bcl-xShort and Bid are known as pro-apoptotic proteins [24,25].

Activated lymphocytes are removed by Fas/FasL mechanism called activation induced cell death (AICD) [26,27]. The molecule CD95 (APO-1/Fas) is a surface antigen detectable on activated lymphocytes. Thus, activated lymphocytes may be able to undergo Fas/Fas-l-mediated apoptosis process independently of the Bcl-2 protein family. The antigen CD95 (APO-1/Fas) belongs to the tumor necrosis factor-receptor family (TNF-R).

It was reported recently that the proportion of peripheral blood Treg cells decreases in preeclampsia, but it is unclear why Treg cells decrease [16–19].

The aim of our study was to evaluate the levels of CD4^+^CD25^+^FoxP3^+^ Treg cells and CD8^+^CD28^+^ T lymphocytes in peripheral blood of patients with pre-eclampsia and healthy pregnant women in the third trimester of normal pregnancy. Furthermore, the aim of the study was to evaluate the surface expressions of CD95 antigen and intracellular expressions of Bcl-2 and Bax proteins in CD4^+^CD25^+^FoxP3^+^ Treg cells of patients with pre-eclampsia and normal third trimester pregnant women.

## 2. Results and Discussion

The population of CD4^+^CD25^+^FoxP3^+^ Treg cells was significantly lower in peripheral blood of patients with pre-eclampsia when compared to healthy normotensive pregnant women in the third trimester of normal pregnancy (*p* < 0.05). The results are presented in the Table 1.

There were no significant differences in the percentages of CD4^+^CD25^+^FoxP3^+^ Treg cells with the expressions of APO-1/FAS (CD95) antigen in peripheral blood of patients with pre-eclampsia and normal third trimester pregnant women. Similarly, the mean fluorescence intensity (MFI) of APO-1/FAS (CD95) antigen on CD4^+^CD25^+^FoxP3^+^ Treg cells did not differ in peripheral blood of patients with pre-eclampsia when compared to the control group. The results are presented in Tables 1 and 2.

The percentages of peripheral blood CD4^+^CD25^+^FoxP3^+^ Treg cells that express Bax protein were similar in both studied groups. However, MFI of Bax protein in CD4^+^CD25^+^FoxP3^+^ Treg cells was significantly higher in peripheral blood of patients with pre-eclampsia when compared to the control group (*p* < 0.01). The results are presented in Tables 1 and 2.

The percentages of peripheral blood CD4^+^CD25^+^FoxP3^+^ Treg cells that express Bcl-2 protein were significantly lower in patients with pre-eclampsia when compared to the control group (*p* < 0.05). Moreover, MFI of Bcl-2 protein in peripheral blood Treg cells of patients with pre-eclampsia was significantly lower when compared to normal third trimester pregnant women (*p* < 0.05). The results are presented in Tables 1 and 2.

The percentages of peripheral blood CD8^+^CD28^+^ T cells did not differ in the group of patients with pre-eclampsia when compared to the healthy pregnant women. However, the MFI of CD28 antigen on CD8^+^ T cells was significantly higher in the group of patients with pre-eclampsia when compared to the control group (*p* < 0.001). The results are presented in Tables 1 and 2.

During physiological pregnancy there are a lot of alterations in the maternal immune system which prevent an inappropriate response against fetal antigens. Many studies confirmed the theory concerning “Th2 phenomenon”, which claimed that during normal pregnancy there is a predominance of Th2 over Th1 type immunity [28]. In the following years “Th2 phenomenon” was revised [29]. In recent studies, it was hypothesized that Treg cells play a crucial role in the maternal tolerance to fetal antigens. This is possible because of their capacity to regulate the activation of allo-reactive T cells [12–15]. It was observed that normal human pregnancy is associated with the expansion of Treg cells [12–15]. On the other hand, in the cases of patients with pre-eclampsia decreased levels of Treg cells were found [16–19].

Although the factors regulating CD4^+^CD25^+^ Treg cells have not been fully defined yet, there are some reports suggesting that estrogens may modify maternal immune response. Polanczyk *et al*. demonstrated on animal models that the treatment with estrogens up-regulates CD4^+^CD25^+^ T cell subset [30]. It was observed that the concentrations of estrogens are lower in pre-eclampsia when compared to physiological pregnancy [31]. Therefore, it seems possible that the population of Treg cells is decreased in that mechanism in pre-eclampsia.

On the other hand, it has been observed that in pre-eclampsia the production of Interleukin-1β (IL-1β) and Interleukin-6 (IL-6) is increased [32]. It is known that the secretion of IL-6, which is a pro-inflammatory cytokine, suppresses the protective immunoregulatory properties of CD4^+^CD25^+^ Treg cells [33]. It seems possible that the increased levels of IL-6 in pre-eclampsia can suppress the expansion of Treg cells in this syndrome.

It has been shown in recent studies that Th17 cells, which induce inflammatory response and Treg cells, which have regulatory properties are developmentally linked [34]. Interleukin-1β and IL-6 induce the differentiation of naïve T cells into Th17 cells, and these cytokines could induce the development of Th17 cells from Treg cells [34,35]. Transforming growth factor-β (TGF-β) is known to drive the differentiation of Treg cells and to inhibit the differentiation of Th17 cells [36]. In pre-eclampsia the differentiation of Treg cells might be decreased resulting in small numbers of Tregs because of elevated levels of soluble endoglin, which is known to inhibit the TGF-β signalling [37]. In fact, Santner-Nanan *et al*. reported in their study that the population of Th17 cells is increased and the population of Treg cells is decreased in pre-eclampsia [18].

Finally, the deficit of Treg cells in pre-eclampsia might be explained by the susceptibility to apoptosis in Treg cells. Fas/FasL-mediated apoptosis seems to be the most important mechanism to keep appropriate balance during pregnancy. Normally, anti-apoptotic protein Bcl-2 remains unchanged during normal pregnancy [37].

It has been noticed that the induction of Fas/Fas-L mechanism can lead to the depletion of Treg lymphocytes [30]. It was found that APO-1/Fas (CD95) antigen is highly expressed on CD4^+^CD25^+^FoxP3^+^ Treg cells. Treg cells are highly susceptible to the ligand for CD95 (CD95L), but not to T cell receptor mediated cell death [38].

The family of Bcl-2 protein is composed of both pro- and anti-apoptotic proteins. It has been suggested that progesterone modulates the expressions of Bcl-2 and Bax proteins. Low concentrations of progesterone lead to the down-regulation of Bax protein and increased levels of Bcl-2 protein [38]. The protein Bcl-2 is described as an inhibitor of programmed cell death, it down-regulates cytochrome c mitochondrial release notably by impairing the pro-apoptotic function of Bax protein. In contrast, the protein Bax releases cytochrome c from mitochondria which induces apoptosis through the activation of caspases 3, 6, 7, 9 [24,25].

In our study we observed the deficit of CD4^+^CD25^+^FoxP3^+^ Treg cells in peripheral blood of patients with pre-eclampsia when compared to healthy normotensive pregnant women in the third trimester of normal pregnancy. We hypothesized that the alterations in apoptotic mechanisms are responsible for decreased levels of Treg lymphocytes in pre-eclampsia. We revealed significantly lower percentages of peripheral blood CD4^+^CD25^+^FoxP3^+^ Treg cells that express Bcl-2 protein as well as significantly lower MFI of Bcl-2 protein and higher MFI of Bax protein in Treg cells of pre-eclamptic patients in comparison with normal third trimester pregnant patients. The results may suggest that apoptosis of activated CD4^+^CD25^+^FoxP3^+^ Treg cells is up-regulated in pre-eclampsia, although we did not show the direct evidence of apoptosis of Treg cells of pre-eclamptic patients. It is very difficult to show the apoptotic peripheral blood cells, because these cells are eliminated by phagocytosis. Indeed, there are no papers that show apoptotic peripheral blood cells in cases of obstetric complications of human pregnancy.

We did not find any differences in the expressions of CD95 (APO-1/Fas) antigen on peripheral blood CD4^+^CD25^+^FoxP3^+^ Treg cells of patients with pre-eclampsia and normal pregnant patients. The results suggest that the deficit of Treg cells in pre-eclampsia is not associated with the alterations in the expressions of CD95 antigen.

In the present study we also evaluated the expressions of CD28 antigen on peripheral blood CD8^+^ T lymphocytes of patients with pre-eclampsia and normal third trimester pregnant women. The antigen CD28 is thought to be a co-stimulatory molecule which may enhance T-cells activation [22,39]. We studied the percentages of T CD8^+^CD28^+^ lymphocytes but we did not find any differences between the populations of these cells in normal pregnancy and pre-eclampsia. However, the mean fluorescence intensity of CD28 antigen on T CD8^+^ lymphocytes was significantly higher in pre-eclampsia when compared to normal third trimester pregnant women. The results suggest the role of CD28 antigen in T cell activation in the pathogenesis of pre-eclampsia.

The crucial influence of CD28 and CTLA-4 (cytotoxic T-lymphocyte antigen-4) antigens on T-cell immune response has been known for over a decade. Initially viewed as molecules that provide intracellular stimulatory and inhibitory signals, recent evidence suggests that both of them are also important in the homeostasis and function of a regulatory T cells (Treg). The antigens CD28 and CTLA-4 play an important role in the development of T cells in thymus and both can influence thymic selection of T cells. They have an impact on positive/negative selection of Treg and CD28 signaling regulates expression of Foxp3 in the thymocytes. These two molecules are also important in generation and regulation of Tregs in peripheral tissues. Tregs may utilize CTLA-4 (or possibly CD28) expression to increase expression of IDO (indoleamine 2,3-dioxygenase ) in dendritic cells (DC). This may exert a dominant suppressive effect by depleting the environment of tryptophan. The antigen CD28 may provide signals for Treg proliferation as well as survival. The antigen CTLA-4 is implicated in the suppressive function of Tregs. The disruption of the interaction of CTLA-4 with its ligands can downregulate the immunosupressive activity of Treg. Treg interactions with DC may alter the adhesion or motility of CD25 negativeT lymphocytes and it relate to a role proposed for CTLA-4 in T-cell adhesion [39].

## 3. Material and Methods

### 3.1. Patients

The patients participating in this study were admitted to the Department of Obstetrics and Perinatology, Medical University of Lublin, Poland. The study group consisted of 24 pregnant women with pre-eclampsia. They were admitted to the hospital because of the symptoms of the disease but not because of signs of labour. Pre-eclamptic patients were classified according to the regulations of the Committee on Terminology of *American College of Obstetricians and Gynecologists*. Pre-eclampsia was characterized by blood pressure of at least 140/90 mmHg and proteinuria above 0.3 g/24 h. None of the pre-eclamptic patients was affected by preexisting clinical disorders, such as: chronic hypertension, renal diseases before pregnancy, and none of the pregnancies was complicated by preterm labor or chorioamnionitis. The control group included 20 healthy normotensive pregnant women in the third trimester of normal pregnancy. They were recruited from the outpatient clinic. All pregnancies from the study and control groups were single. The patients from the study and control groups were not in active labor. The characteristics of the study and control groups are presented in Table 3. The study design was accepted by the local Ethics Committee. Informed consent for peripheral blood sampling was obtained from the patients.

### 3.2. Blood Sampling and Cell Preparation

Twenty milliliters of blood from both pre-eclamptic patients and normal third trimester pregnant women were taken by venipuncture and collected in sterile heparinized tubes. Blood samples from the patients with pre-eclampsia were taken before any treatment, such as: the administration of antenatal steroids or antihypertensive drugs.

### 3.3. Isolation of Peripheral Blood Cells

Peripheral blood mononuclear cells (PBMCs) were separated by gradient centrifugation on the lymphocyte separation medium, Lymphoprep (Nycomed, Torshov, Norway). They were centrifuged for 30 min at 600× g at 4 °C, collected from the interface with a Pasteur pipette and washed twice by centrifugation for 5 min at 250 g at 4 °C in 2 mL of the buffer containing phosphate-buffered saline (PBS; Serum and Vaccine Factory, Biomed, Lublin, Poland) with 2% FCS (Fetal Calf Serum, GibcoBRL, Paisley, UK) and the total numbers of cells were determined using the microscope.

### 3.4. Phenotyping of T Cells

The cell surface and intracellular antigens were determined on the fresh cells at the time of the sample submission. The cells were stained according to the manufacturer’s protocols. The cells were labeled by direct staining of monoclonal antibodies. Five hundred microliters of cell suspensions were added to 5 μL of appropriate solution of fluorescein isothiocyanate (FITC)-conjugated antibodies (Dako, Denmark and Becton Dickinson, San Diego, CA, USA), phycoerythrin (PE)-conjugated antibodies (BioLegend, San Diego, CA, USA), PE-Cy5-conjugated antibodies (BioLegend, San Diego, CA, USA) and Pacific Blue-labeled antibodies (BioLegend, San Diego, CA, USA) in the following combinations:

1. anti-Human CD8 (FITC) and anti-Human CD28 (PE) monoclonal antibodies;
2. anti-Human CD95 (FITC), anti-Human CD25 (PE), anti-Human CD4 (PE-Cy5) and anti-Human FoxP3 (Pacific Blue) monoclonal antibodies;
3. anti-Human Bax (FITC), anti-Human CD25 (PE), anti-Human CD4 (PE-Cy5) and anti-Human FoxP3 (Pacific Blue) monoclonal antibodies;
4. anti-Human Bcl-2 Oncoprotein (FITC), anti-Human CD25 (PE), anti-Human CD4 (PE-Cy5) and anti-Human FoxP3 (Pacific Blue) monoclonal antibodies.

**Figure 1:**
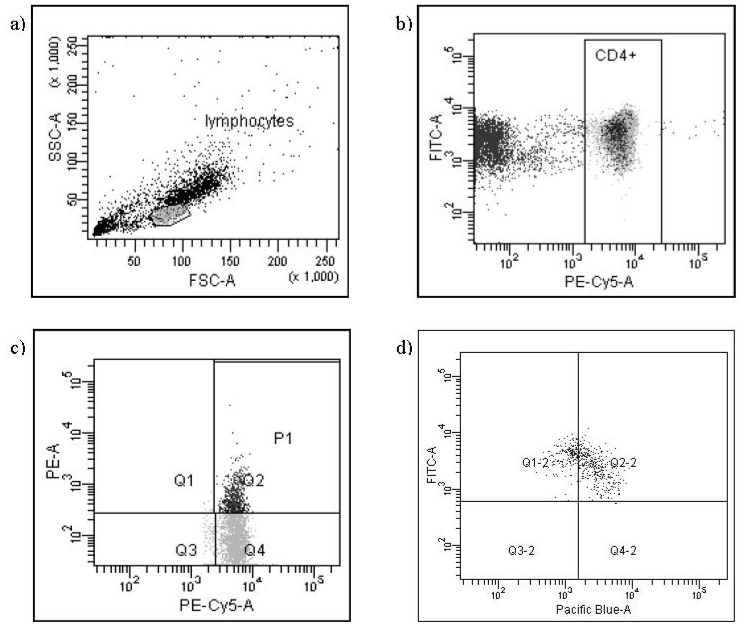
Flow cytometry analysis of the Bcl-2 antigen expression in CD4^+^CD25^+^FoxP3^+^ cells. FITC—anti-Bcl-2, PE—anti-CD25, PE-Cy5—anti-CD4, Pacific Blue—anti-FoxP3. (**a**) gating of lymphocytes; (**b**) gating of CD4^+^ cells; (**c**) gating of CD4^+^CD25^+^ cells; (**d**) analysis of Bcl-2^+^CD4^+^CD25^+^FoxP3^+^ (Q2-2) and Bcl-2^+^CD4^+^CD25^+^FoxP3^−^ (Q1-2).

**Figure 2:**
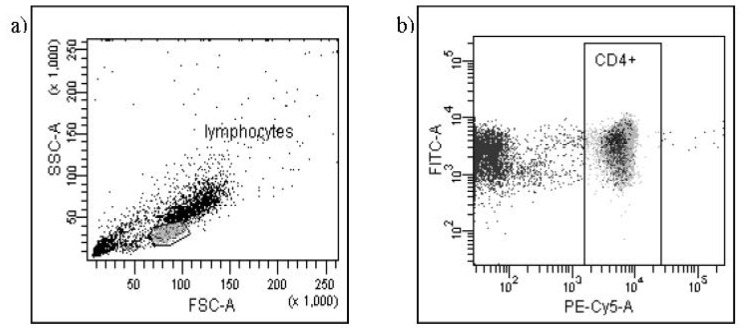

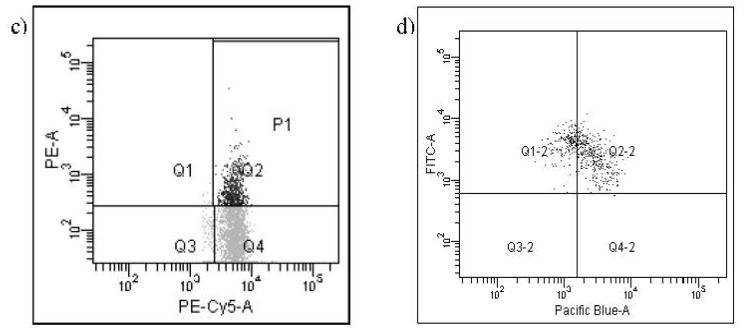
Flow cytometry analysis of the Bax antigen expression in CD4^+^CD25^+^FoxP3^+^ cells. FITC—anti-Bax, PE—anti-CD25, PE-Cy5—anti-CD4, Pacific Blue—anti-FoxP3. (**a**) gating of lymphocytes; (**b**) gating of CD4^+^ cells; (**c**) gating of CD4^+^CD25^+^ cells; (**d**) analysis of Bax^+^CD4^+^CD25^+^FoxP3^+^ (Q2-2) and Bax^+^CD4^+^CD25^+^FoxP3^−^ (Q1-2).

The mixture of cells and antibodies was incubated for 30 min at 4 °C in the dark, centrifuged, washed twice by adding 1 mL of cold PBS to each tube with 1% sodium azide and 1% FCS and centrifuged again at 400× g for 10 min. After the standard incubation with antibodies directed against surface markers, the incubation by fixation and permeabilisation with FoxP3 Fix/Perm Buffer and FoxP3 Perm Buffer (BioLegend, San Diego, CA, USA) was performed. The next step of the protocol was incubation with antibodies directed against intracellular proteins: FoxP3 and Bcl-2 or Bax. Next, the supernatant was separated and after washing each sample was suspended in 200 μL PBS. As the last step, cells were immediately analyzed with the use of Becton Dickinson Canto II flow cytometer (Becton Dickinson, San Diego, CA, USA) and analyzed with FACSDiva™ Software (Becton Dickinson, San Diego, CA, USA). The results were presented as percentage of cells stained with antibody. The percentage of positive cells was calculated by comparing with the control. Background fluorescence was determined using isotype-matched directly conjugated mouse anti IgG1/IgG2α monoclonal antibodies. The samples were gated on forward scatter versus side scatter to exclude debris and cell aggregates. To assess the number of places binding a monoclonal antibody and indirectly the number of antigens the Mean Fluorescence Intensity (MFI) was used. For the assessment of MFI, logarithmic units were applied with the use of FACSDiva™ Software (Becton Dickinson, San Diego, CA, USA). The Mean Fluorescence Intensity was measured in comparison to the upper limit of MFI of negative control. The representatives FACS plots of the expressions of Bcl-2 and Bax protein in T CD4^+^CD25^+^FoxP3^+^ Treg cells in peripheral blood of one patient with pre-eclampsia are presented in Figures 1 and 2.

### 3.5. Statistical Analysis

Statistical differences between groups were estimated using a standard non-parametric test (Mann-Whitney U-test). The results were presented as a median and interquartile ranges. Differences were defined as statistically significant at the level of *p* < 0.05. Statistica 7.0 software (StatSoft Poland, Krakow, Poland) was applied to statistical analysis.

## 4. Conclusions

The obtained results suggest that the deficit of CD4^+^CD25^+^FoxP3^+^ Treg lymphocytes which is observed in pre-eclampsia may be associated with altered apoptosis signaling in Tregs.

## Figures and Tables

**Table 1:** The percentage of CD4^+^CD25^+^FoxP3^+^ Treg cells as well as the expressions of CD95, Bax and Bcl-2 antigens on CD4^+^CD25^+^FoxP3^+^ Treg cells in peripheral blood of patients with pre-eclampsia and health third trimester pregnant women.

	Patients with pre-eclampsia (*n* = 24) median and interquartile ranges	Normal third trimester pregnancy (*n* = 20) median and interquartile ranges	*p*
CD4^+^CD25^+^FoxP3^+^ Treg cells	3.60% (2.30%–6.01%)	6.20% (5.15%–7.60%)	*p* < 0.05
CD95 antigen on CD4^+^CD25^+^FoxP3^+^ Treg cells	63.34% (49.38%–68.60%)	64.32% (51.90%–72.89%)	NS
Bcl-2 protein on CD4^+^CD25^+^FoxP3^+^ Treg cells	69.10% (52.00%–88.00%)	97.45% (95.70%–99.30%)	*p* < 0.05
Bax protein on CD4^+^CD25^+^FoxP3^+^ Treg cells	21.15% (19.59%–28.95%)	23.97% (19.77%–30.66%)	NS
CD8^+^CD28^+^ T cells	6.50% (3.59%–12.85%)	6.75% (4.07%–9.41%)	NS

**Table 2:** The mean fluorescence intensity (MFI) of CD95, Bax and Bcl-2 antigen on CD4^+^CD25^+^FoxP3^+^ Treg cells as well as MFI of CD28 antigen on T CD8^+^ cells in peripheral blood of patients with pre-eclampsia and healthy third trimester pregnant women.

	Patients with pre-eclampsia (*n* = 24) median and interquartile ranges (arbitral units)	Healthy third trimester pregnant women (*n* = 20) median and interquartile ranges (arbitral units)	*p*
MFI CD95 antigen on CD4^+^CD25^+^FoxP3^+^ cells	52.05 a.u. (46.93 a.u.–56.62 a.u.)	49.41 a.u. (42.79 a.u.–55.60 a.u.)	NS
MFI Bax antigen on CD4^+^CD25^+^FoxP3^+^ cells	185.34 a.u. (150.51 a.u.–206.13 a.u.)	102.76 a.u. (86.13 a.u.–124.15 a.u.)	*p* < 0.01
MFI Bcl-2 antigen on CD4^+^CD25^+^FoxP3^+^ cells	2343 a.u. (2077.00 a.u.–2592 a.u.)	2800.00 a.u. (2315.00 a.u.–3858.00 a.u.)	*p* < 0.05
MFI CD28 antigen on T CD8^+^ cells	131.26 a.u. (98.47 a.u.–146.14 a.u.)	63.34 a.u. (68.56 a.u.–88.95 a.u.)	*p* < 0.001

**Table 3:** The clinical characteristics of patients with pre-eclampsia (study group) and healthy normotensive women in the third trimester of uncomplicated pregnancy (control group).

	Study group mean ± SD *n* = 24	Control group mean ± SD *n* = 20	Significance (*p*)
Maternal age	28.68 ± 4.76	27.68 ± 5.31	NS
Gravidity	1.84 ± 1.12	1.93 ± 0.85	NS
Parity	1.63 ± 0.95	1.81 ± 0.81	NS
Time of blood collection (weeks of gestation)	34.05 ± 2.14	34.62 ± 1.63	NS
Systolic pressure (mmHg)	155.35 ± 15.85	110.25 ± 20.15	<0.01
Diastolic pressure (mmHg)	96.47 ± 5.66	73.56 ± 7.18	<0.05
Proteinuria (g/24 h)	1.45 ± 0.65	absent	-
Uric acid (mg/dL)	5.47 ± 1.38	3.15 ± 1.43	<0.01
Fetal weight (g)	2560 ± 615	3280 ± 365	<0.05
